# Investigating the molecular genetic, genomic, brain structural, and brain functional correlates of latent transdiagnostic dimensions of psychopathology across the lifespan: Protocol for a systematic review and meta-analysis of cross-sectional and longitudinal studies in the general population

**DOI:** 10.3389/fpsyt.2022.1036794

**Published:** 2022-11-03

**Authors:** Nicholas Hoy, Samantha Lynch, Monika Waszczuk, Simone Reppermund, Louise Mewton

**Affiliations:** ^1^Centre for Healthy Brain Ageing, University of New South Wales, Sydney, NSW, Australia; ^2^The Matilda Centre for Research in Mental Health and Substance Use, University of Sydney, Sydney, NSW, Australia; ^3^Department of Psychology, Rosalind Franklin University of Medicine and Science, North Chicago, IL, United States; ^4^Department of Developmental Disability Neuropsychiatry, University of New South Wales, Sydney, NSW, Australia

**Keywords:** psychopathology, p-factor, internalising, externalising, genomic, brain structure, brain function, lifespan

## Abstract

**Background:**

Research using latent variable modelling has identified a superordinate general dimension of psychopathology, as well as several specific/lower-order transdiagnostic dimensions (e.g., internalising and externalising) within the meta-structure of psychiatric symptoms. These models can facilitate discovery in genetic and neuroscientific research by providing empirically derived psychiatric phenotypes, offering greater validity and reliability than traditional diagnostic categories. The prospective review outlined in this protocol aims to integrate and assess evidence from research investigating the biological correlates of general psychopathology and specific/lower-order transdiagnostic symptom dimensions. Cross-sectional and longitudinal studies investigating general population samples of any age group or developmental period will be included to capture evidence from across the lifespan.

**Methods and analysis:**

MEDLINE, Embase, and PsycINFO databases will be systematically searched for relevant literature. The review will follow the Preferred Reporting Items for Systematic Reviews and Meta-Analyses (PRISMA) guidelines. Eligibility criteria were designed to capture psychiatric genetic (i.e., molecular genetic and genomic) and neuroimaging (i.e., brain structural and brain functional) studies investigating latent transdiagnostic dimension(s) or structural model(s) of psychopathology across any age group. Studies which include or exclude participants based on clinical symptoms, disorders, or relevant risk factors (e.g., history of abuse, neglect, and trauma) will be excluded. Biometric genetic research (e.g., twin and family studies), candidate gene studies, neurophysiology studies, and other non-imaging based neuroscientific studies (e.g., post-mortem studies) will be excluded. Study quality and risk of bias will be assessed using the Joanna Briggs Checklist for Analytical Cross-Sectional Studies, the Joanna Briggs Checklist for Cohort Studies, and the Grades of Recommendation, Assessment, Development, and Evaluation (GRADE) system. Meta-analysis will be conducted if sufficient data is available.

**Discussion:**

This protocol outlines the first systematic review to examine evidence from studies investigating the latent structure and underlying biology of psychopathology and to characterise these relationships developmentally across the lifespan. The prospective review will cover a broad range of statistical techniques and models used to investigate latent transdiagnostic dimensions of psychopathology, as well as a numerous genetic and neuroscientific methods.

**Systematic review registration:**

[https://www.crd.york.ac.uk/prospero/], identifier[CRD42021262717].

## Introduction

Psychiatric genetic and neuroscientific research informs our understanding of the aetiology, course, and consequences of mental illness, improves diagnostic accuracy, and guides the development of effective and biologically informed preventative interventions, and treatment strategies. Over the past three decades, significant advances in genetic sequencing and neuroimaging brought the promise of ushering in an unprecedented era of discovery in biological psychiatry (1–3). However, despite these methodological developments, researchers have made little progress in identifying clinically useful biomarkers for different psychiatric disorders or in reducing the burden of mental illness in the general population (3, 4). A growing consensus among researchers is that this lack of progress has been driven by reliance on the categorical model of psychopathology in psychiatric research, which is increasingly recognised to provide suboptimal phenotypes through which to investigate the biological underpinnings of mental illness (5–9).

### Latent variable models of psychopathology

As an alternative to the categorical approach, latent variable models of dimensional psychopathology have been proposed. Seminal factor-analytic studies demonstrated that two latent transdiagnostic dimensions of psychopathology (i.e., internalising and externalising) could be extracted from patterns of covariation across a range of common psychiatric disorders (10, 11). The internalising dimension typically captures more emotionally focused symptoms, maladaptive traits and/or disorders (e.g., depression, anxiety, and specific phobia), whereas the externalising dimension captures those that are more behaviourally focused (e.g., substance use, inattention, and aggression) (6, 12). Thus, closely related indicators of supposedly distinct expressions of psychopathology (e.g., of depression and anxiety) are assigned to a given higher-order symptom dimension (e.g., internalising), which reflects the patterns of comorbidity between them. Subsequent research, which included broader measurement of psychopathology, identified an additional thought disorder dimension capturing more psychotic like symptoms (e.g., delusions, hallucinations, and disorganised thought) (13, 14). Several other latent transdiagnostic dimensions of psychopathology have since been identified and some (e.g., internalising and externalising) have also been found to bifurcate into additional subfactors (15).

Importantly, significant positive correlations among these latent dimensions led to the identification of a superordinate general dimension of psychopathology (often referred to as the p-factor) (16). This general dimension of psychopathology suggests that the meta-structure of mental illness can be understood hierarchically, including both a *single* general symptom dimension, as well as several specific/lower-order transdiagnostic symptom dimensions (e.g., internalising, externalising, and thought disorder) that sit at lower levels of the hierarchy. General psychopathology is argued to reflect an underlying liability to develop any and all manifestations of psychopathology (17, 18); however, the validity and substantive meaning of this dimension is the subject of ongoing debate (19, 20). Researchers have advanced several theories as to the substantive meaning of general psychopathology, including that it reflects negative emotionality (21), impaired emotional regulation (22), disordered thought processes (17), and functional impairment (23).

### Advantages of latent transdiagnostic dimensional models in genetic and neuroscientific research

Latent transdiagnostic dimensional models can advance our understanding of the genetic and neural correlates of psychopathology. For one, dimensional phenotypes offer greater precision and statistical power than traditional diagnostic categories, facilitating discovery in genetic and neuroscientific research (7, 8). For example, traditional case-control studies impose arbitrary symptom thresholds in selecting cases and thereby suffer from considerable loss of information with respect to variations in symptom severity (e.g., subthreshold cases) (7). By contrast, dimensional phenotypes capture the full range of symptom severity, allowing for more precise estimates of a given association between biology and symptom expression.

Another advantage of the latent variable approach is that it allows for directly modelling the observed correlational structure and dimensionality of psychiatric symptoms, providing more valid and reliable phenotypes than traditional disorder categories (15). Indeed, accumulating evidence indicates that the biological correlates of psychopathology are largely consistent with the latent hierarchical structure identified through phenotypic research (7, 8, 24). For instance, genome-wide association studies (GWAS) have consistently demonstrated evidence of widespread pleiotropy across different diagnostic categories (25, 26). That is, genes influencing the expression of psychopathology are largely shared across different psychiatric disorders, consistent with the observed correlational structure of psychiatric symptoms. Similarly, recent meta-analytic research has demonstrated evidence of shared abnormalities in both brain structure and function across a range of common psychiatric disorders (27, 28). There is also evidence that the genetic and neural mechanisms underlying different manifestations of psychopathology are associated with trait-like, subclinical manifestations of psychopathology in the general population, supporting the dimensionality of psychiatric symptoms (29, 30). Examining evidence from studies that *directly* investigate the underlying biology of transdiagnostic, hierarchically defined symptom dimensions should, therefore, enhance our understanding of the relationship between genetics, neurobiology, and mental illness.

Lastly, latent dimensional models allow for investigating the biological correlates of psychopathology at various levels of specificity and across the full range of phenotype severity (7, 8, 24). Researchers can target genetic and neural mechanisms that are associated with broad, non-specific manifestations of psychopathology, as well as those associated with specific/lower-order dimensions and subdimensions. Importantly, identifying biological correlates that are distinctly and consistently associated with general psychopathology and specific/lower-order symptom dimensions is critical to supporting the validity of the hierarchical model, as well as its utility in genetic and neuroscientific research (24). As such, the upcoming review will integrate and assess evidence from studies investigating the genetic (i.e., molecular genetic and genomic) and neural (i.e., brain structural and brain functional) correlates of latent transdiagnostic phenotypes at multiple levels of specificity (i.e., general psychopathology and specific/lower-order transdiagnostic symptom dimensions) and evaluate whether there is evidence of distinct and replicable biological correlates at different levels of the symptom hierarchy.

### Investigating the latent structure and biological correlates of psychopathology across the lifespan

The latent hierarchical structure of psychopathology has been replicated across different age groups and developmental periods, from early childhood through to older adulthood (21). However, research to date has primarily been conducted using cross-sectional samples of adults (15). Consequently, important gaps exist in our understanding of the onset and developmental course of different symptom dimensions and of the biological factors driving differences in the trajectories of these dimensions across the lifespan (15, 21). At the level of categorical diagnoses, research has long demonstrated evidence of age- and developmentally specific patterns in the onset and course of psychiatric disorders, as well as periods associated with both increased and decreased risk of mental illness (31). These trajectories are driven by genetic, neurobiological, and environmental factors that precede the onset of psychopathology, as well as both normative and non-normative changes in gene expression, neurobiology and environmental risk that occur across the lifespan (9, 31–33). Disentangling the temporal ordering of associations between genetics, neurobiology, and symptom expression across different age groups and developmental periods is therefore critical to accurately modelling the structure and biological underpinnings of psychopathology. From a clinical perspective, this research is of paramount importance because it guides the development of biologically informed preventative and early intervention efforts (e.g., by identifying biomarkers that predict the onset of psychopathology), as well as the development of effective treatment strategies (e.g., by identifying biomarkers associated with active psychopathology, or prolonged exposure to psychopathology, which provide targets for clinical and pharmacological intervention) (34).

As such, the upcoming review will integrate existing evidence from studies investigating transdiagnostic symptom dimensions and their underlying biology in any age group in order to assess evidence from across the lifespan. Cross-sectional studies will be included to integrate and assess evidence of the molecular genetic, genomic, brain structural, and brain functional mechanisms and processes which correlate with different latent dimensional phenotypes age-specifically and across age groups. Longitudinal research will be included to integrate and assess evidence regarding the temporal ordering of associations between genetics, neurobiology, and the onset and course of latent dimensional phenotypes across different timeframes and developmental periods. In addition, the review will highlight priority (i.e., understudied and promising) areas for future genetic and neuroimaging research investigating latent dimensional phenotypes across different age groups and developmental periods.

While there are several published reviews examining evidence from studies investigating the latent structure and underlying biology of psychopathology (6–9, 15, 35), none of these reviews were conducted systematically and those which focused specifically on genetic and neuroimaging research (7–9) included only a select number of studies directly investigating the biological correlates of different latent dimensional phenotypes. One systematic review has examined evidence of risk and protective factors (including biological factors) associated with general and specific latent symptom dimensions (36). However, this review was restricted to samples of youth aged 10–24 years old, characterising only a narrow (albeit highly important) developmental period. The upcoming review will extend these findings by systematically reviewing evidence from across the lifespan, providing a more comprehensive examination of evidence from studies investigating the biological correlates of general and specific/lower-order dimensions of psychopathology.

### The prospective review

The current paper outlines the protocol for an upcoming systematic review aiming to:

1. Integrate and assess evidence from cross-sectional and longitudinal studies investigating the molecular genetic, genomic, brain structural, and brain functional correlates of general psychopathology and specific/lower-order symptom dimensions across the lifespan in the general population.
2. Determine whether there is evidence of distinct genetic and/or neural correlates that are associated with general psychopathology and specific/lower order transdiagnostic symptom dimensions.
3. Determine whether there is evidence of age-related differences in the genetic and neural correlates of general psychopathology and specific/lower-order transdiagnostic symptom dimensions.

A systematic literature review will be conducted to identify cross-sectional and longitudinal studies investigating the genetic (i.e., molecular genetic and genomic) and neural (i.e., brain structural and brain functional) correlates of latent transdiagnostic dimensions of psychopathology in the general population [e.g., (37, 38)]. Studies investigating any age group or developmental period will be included to integrate evidence from across the lifespan. Studies investigating any latent transdiagnostic dimension(s) (e.g., general psychopathology, internalising, externalising, and thought disorder) or latent structural model(s) (e.g., bifactor models and hierarchical models) will be included to capture evidence of shared and distinct biological correlates associated with transdiagnostic symptom dimensions across multiple levels of specificity. This will be the first systematic literature review to specifically investigate the molecular genetic, genomic, brain structural, and brain functional correlates of latent transdiagnostic dimensions of psychopathology and the first to characterise these relationships systematically across the lifespan.

## Methods

### Study design

This protocol was developed in accordance with the Preferred Reporting Items for Systematic Reviews and Meta-Analysis Protocols (PRISMA-P) statement (see Supplementary Table 1) (39). The protocol has been registered with the International Prospective Register of Systematic Reviews (PROSPERO; registration number: CRD42021262717). Any amendments made to the protocol will be documented through PROSPERO.

### Search strategy

A comprehensive search strategy will be used to identify relevant literature from three different electronic databases (i.e., Embase, MEDLINE, and PsycINFO). The search strategy is provided in the Supplementary material (see Supplementary Tables 2–4). Relevant literature will be searched across each database, with no additional restrictions imposed on the date, language, or type of publication. The reference lists of all included articles and relevant reviews will be manually searched for additional citations. Each search strategy includes a broad combination of relevant database-specific subject headings and additional keywords, developed by identifying terms used to index highly relevant papers across the three databases and by adapting the search terms of previous systematic literature reviews that examined latent dimensional models of psychopathology (21, 36). Search strings will be adapted for each database, given that they each index papers according to different subject-headings (40). The search strategy captures three major domains: latent variable models of psychopathology; molecular genetic and genomic research; and neuroimaging research. The overall search strategy, combining each different domain, functions as follows: (latent variable model terms AND psychopathology terms) AND (molecular genetic OR genomic research terms), OR (brain structural OR brain functional neuroimaging research terms).

### Eligibility criteria

The research questions and eligibility criteria for the review were developed using the Population Exposure Comparator Outcome Study (PECOS) framework (41).

### Inclusion criteria

#### Population

1. Only studies investigating general population samples will be eligible for inclusion.
2. Studies investigating any age group will be eligible.
3. Only studies investigating human participants will be eligible.

### Exposure

1. Studies using any latent variable modelling technique (e.g., factor analysis, principal component analysis, and structural equation modelling) to investigate latent transdiagnostic psychiatric phenotypes as the exposure will be eligible for inclusion.
  a. Studies investigating any latent transdiagnostic dimension(s) of psychopathology (e.g., general psychopathology, internalising, externalising, and thought disorder) will be eligible.
  b. Studies investigating any latent structural model(s) of psychopathology (e.g., bifactor models, hierarchical models, and correlated factor models) will be eligible.
2. Studies using any technique to investigate molecular genetic or genomic variables as the exposure (with the exception of candidate gene studies) will be eligible for inclusion.
3. Studies using any neuroimaging technique to investigate any brain structural or brain functional variable as the exposure will be eligible for inclusion.
4. Both whole-brain and region of interest neuroimaging studies will be eligible.

#### Comparator (not applicable)

##### Outcomes

1. For studies that treat psychiatric phenotypes as the exposure, the outcome measure must include at least one biological variable (i.e., molecular genetic, genomic, brain structural, and/or brain functional).
2. For studies that treat biological variables as the exposure, at least one latent transdiagnostic dimension of psychopathology (e.g., general psychopathology, internalising, and externalising) must be measured as the outcome.
3. Only studies reporting empirical data will be included.

##### Study characteristics

1. Only peer-reviewed studies will be included.
2. Both cross-sectional and longitudinal studies will be eligible.
3. Studies including any sample size will be eligible.
4. Studies written in any language will be eligible.

### Exclusion criteria

#### Population

1. Studies in which participants were included or excluded based on clinical symptoms, psychiatric disorders, or relevant risk factors (e.g., history of abuse, neglect, or maltreatment) will be excluded.
2. Studies of non-human animals will be excluded.

#### Exposures/outcomes

1. Studies investigating specific symptom (i.e., first order) dimensions or any other latent variable that does not capture transdiagnostic psychopathology (i.e., that does not include indicators from across different psychiatric disorder categories) as either the exposure or outcome will be excluded.
2. Studies in which transdiagnostic dimensional measures of psychopathology are treated as the exposure or outcome but are not measured using latent variable techniques (e.g., total scores on instruments with broad measurement of psychopathology) will be excluded.
3. Studies that include biometric genetic measures (e.g., twin, family, and adoption studies) will be excluded.
4. Candidate gene studies will be excluded.
5. Neurophysiological studies (e.g., studies using electroencephalography to measure neural activity) will be excluded.
6. Neuroscientific studies using techniques other than neuroimaging (e.g., post-mortem studies) will be excluded.

#### Study characteristics

1. Publications that do not report peer-reviewed research (e.g., grey literature and conference abstracts) or original empirical findings (e.g., reviews, opinion pieces, letters, books, or book chapters) will be excluded.

### Population

As the review aims to capture evidence from across the lifespan, studies investigating human participants of all age groups will be eligible for inclusion. The review will only include studies using general population samples. Any study in which participants were included or excluded based on clinical symptoms, psychiatric disorders, or relevant risk factors (e.g., history of abuse, neglect, or trauma) will not be eligible. Although studies using clinical samples provide important evidence (e.g., identifying biological correlates that are distinctly associated with greater symptom severity), including studies of both general population and clinical samples is beyond the scope of the upcoming review. Studies investigating general population samples were chosen over studies of clinical samples for the following reasons: (1) general population samples capture the full distribution of psychopathology (compared to clinical samples, which capture only the more severe end of this distribution); (2) the results are more generalisable than the results of studies investigating clinical samples; and (3) general population samples are more commonly studied in the relevant literature than clinical samples. Of note, studies in which participants were selected on the basis of characteristics not related to psychopathology or relevant risk factors (e.g., studies of university students, particular age groups, or particular genetic ancestries) will be eligible for inclusion.

### Exposures/outcomes

**FIGURE 1:**
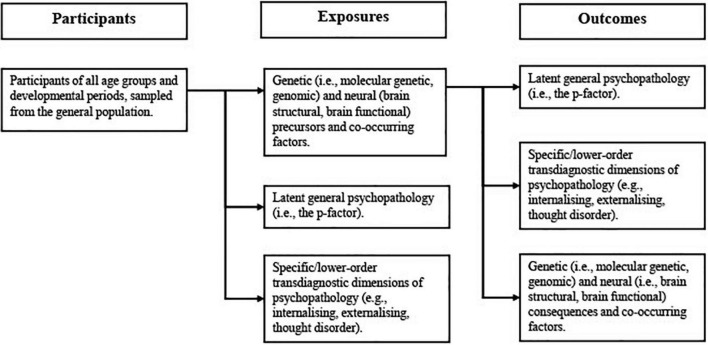
Exposure/outcome relationships that will be examined in the upcoming review.

Given the bidirectional nature of many relationships between psychiatric symptoms, genetics, and neurobiology (31), the review will include studies that treat either psychopathology (i.e., latent transdiagnostic dimensional phenotypes) or the biological correlates of psychopathology (i.e., molecular genetic, genomic, brain structural, or brain functional) as the exposure (see Figure 1). For studies treating psychiatric phenotypes as the exposure, at least one biological variable (i.e., molecular genetic, genomic, brain structural, or brain functional) must be measured as the outcome. For studies treating biological variables as the exposure, at least one latent transdiagnostic dimension of psychopathology (e.g., general psychopathology, internalising, externalising, and thought disorder) must be measured as the outcome. Whether latent dimensional phenotypes were treated as the exposure or outcome will be discussed, as will the implications that this has for the findings of each study (e.g., the effect of psychopathology on brain structure or the effect of brain structure on psychopathology). All outcomes will also be assessed with reference to the latent variable models used to extract different dimensional phenotypes and the implications that this has for the interpretation of the results. (42). The review will provide detailed summaries of significant findings, evaluate the quality of the analysis methods and outcome measures used, as well as the timing of outcome measurement.

The review will include studies investigating a wide range of latent dimensional phenotypes as either exposure or outcome. Studies using any indicators/measures (e.g., self-report, informant-report, and clinician-rated measures) of psychopathology, any latent variable techniques (e.g., factor analysis, principal component analysis, and structural equation modelling), and any model (e.g., bifactor, hierarchical, correlated factor, and single factor) to extract transdiagnostic dimensional phenotypes will be eligible. Studies using latent class analysis or hybrid models that measure psychopathology as a transdiagnostic, dimensional construct will also be eligible. Studies investigating general psychopathology (i.e., the p-factor); individual specific/lower-order dimensions (e.g., internalising and externalising) and sub-dimensions (e.g., disinhibited externalising, antagonistic externalising, fear, and distress); as well as studies investigating multiple dimensions simultaneously (e.g., hierarchical models and correlated factor models) will all be eligible for inclusion. Studies investigating first-order symptom dimensions will be excluded. Studies that use non-latent measures of transdiagnostic dimensional phenotypes (e.g., total scores on instruments with broad measurement of psychopathology) will also be excluded.

The review will also include a wide range of biological variables, treated as either exposure or outcome. Molecular genetic and genomic research studies will be eligible for inclusion, encompassing a wide range of variables and methods used in genetic research (e.g., polygenic risk scores and genomic structural equation modelling). Studies using biometric genetic methods (i.e., family, twin, and adoption studies) are beyond the scope of the review and will not be included. Candidate gene studies, which are widely considered obsolete and unlikely to produce replicable findings in psychiatric genetics (43, 44), will also be excluded from the review. All structural and functional neuroimaging studies will be eligible for inclusion, encompassing a similarly wide range of variables (e.g., white matter integrity and grey matter volume) and methods (e.g., structural and functional magnetic resonance imaging and diffusion tensor imaging) used in neuroscientific research. The review will also include both whole-brain and region of interest neuroimaging studies. Neurophysiological studies (e.g., studies measuring neural activity *via* electroencephalography) and other neuroscientific studies that do not use imaging-based techniques (e.g., post-mortem studies) are beyond the scope of the review and will not be included.

### Comparators

As studies investigating latent variable models of psychopathology preclude the need for a control group, no criteria are necessary for this component of the PECOS framework.

### Study characteristics

Only peer-reviewed research will be eligible for inclusion in the review. Both cross-sectional and longitudinal study designs will be eligible. No minimum sample size restrictions will be imposed; however, sample size will be considered when assessing the methodological quality of included studies. Studies written in a language other than English will be included where possible. Studies of non-human animals will be excluded. Grey literature and conference abstracts will also be excluded, as will reviews, opinion pieces, letters, books, and any other publications that do not report peer-reviewed research or original empirical findings.

### Selection procedure

Two reviewers (i.e., NH and SL) will be involved in screening and study selection procedures for the review. Following de-duplication, reviewer one (NH) will screen all titles and abstracts from across the three databases to identify eligible studies. Reviewer two (SL) will independently screen a random selection of 25% of the titles and abstracts to ensure accuracy of study selection. Cohen’s kappa will be calculated to measure inter-rater agreement between the two reviewers, with a high level of agreement defined as a Cohen’s kappa of 0.80 or above (45). Following title and abstract screening, the full-texts of all included articles will be screened by both reviewers to further assess study eligibility. Cohen’s kappa will also be calculated to determine inter-rater agreement following full-text screening. Disagreements at any stage of the screening process (i.e., title and abstract or full-text) will be resolved through consultation among the two reviewers. If disagreements cannot be resolved, a third member of the research team (i.e., LM, SR, or MW) will be consulted to reach consensus. A PRISMA flowchart will be used to display results from each stage of the screening process.

### Data extraction

All citations will be imported to Covidence (46) for title, abstract and full-text screening. Study data will be extracted independently by NH using a data extraction spreadsheet developed by the research team. NH will pilot the spreadsheet using a random selection of included studies. Study authors will be contacted in the event that any necessary data has not been reported. Specific details about the types of data to be extracted from included studies is provided in Table 1.

**TABLE 1:** Data to be extracted from all included studies.

Type of data	Details
Study information	Name of author(s); year of publication; country in which data was collected.
Study characteristics	Name of study/dataset; study design (i.e., cross-sectional or longitudinal); follow-up details for longitudinal studies (i.e., number of follow-ups and time between follow-ups); setting (i.e., population-based and community-based); research domain (i.e., molecular genetics, genomics, brain structural, and brain functional); sample size.
Participant characteristics	Age (i.e., range and mean); age at each wave (for longitudinal studies); sex (i.e., proportion male and female); nationality, race, and ethnicity.
Psychopathology data	Assessment of psychopathology (e.g., measurement instruments and diagnostic criteria); type of indicators used (e.g., symptom-level, trait-level, joint symptom and trait level, and disorder-level); mode(s) of assessment (e.g., self-report, informant-report, and clinician-rated); dimensions of psychopathology (e.g., general psychopathology, internalising, externalising, and thought disorder); statistical methods for modelling psychopathology (e.g., confirmatory factor analysis and principal components analysis); type of model(s) (e.g., bi-factor, correlated-factors, and hierarchical).
Genetic data	Type of molecular genetic or genomic variables (e.g., single nucleotide polymorphisms); genetic methods and analysis techniques (e.g., polygenic risk scores, genomic SEM).
Neuroimaging data	Neuroimaging focus (e.g., region of interest and whole-brain); neuroimaging techniques (e.g., structural MRI, resting-state fMRI and task-based fMRI, and diffusion tensor imaging); type of task for task-based fMRI; neuroimaging variables (e.g., grey matter volume and white matter integrity).
Outcome data	Analysis methods; test statistics; *p*-values; measures of association (e.g., *R*^2^); reported effect sizes; covariates included in analysis; reported interactions; summary of main findings.

SEM, structural equation modelling; MRI, magnetic resonance imaging; fMRI, functional magnetic resonance imaging.

### Data synthesis

The results of all included genetic (i.e., molecular genetic and genomic) and neuroscientific (i.e., brain structural and brain functional) studies will be reported separately. Tables will report information about: study characteristics (i.e., study design, age, and gender); psychopathology exposure or outcome variables (e.g., general psychopathology and specific/lower-order transdiagnostic symptom dimensions); statistical methods (i.e., factor analysis, principal components analysis, and structural equation modelling) and models (i.e., bi-factor, correlated factor, and hierarchical models) used to measure latent symptom dimensions; biological exposure or outcome variables (i.e., molecular genetic, genomic, brain structural, and brain functional); methods used to measure biological variables; outcome statistics (e.g., measures of effect and effect sizes); and a summary of the main findings.

If sufficient data is available, meta-analyses will be conducted to examine the genetic (i.e., molecular genetic and genomic) and/or neural (i.e., brain structural and brain functional) correlates of latent psychiatric phenotypes (e.g., general psychopathology, internalising, and externalising). Subgroup analyses will investigate whether results vary by age and sex. Sensitivity analyses will assess the impact of study quality. The meta-analysis will be conducted in accordance with evidence-based recommendations for quantitative synthesis of observational studies and separate analyses will be performed for cross-sectional and longitudinal studies (47, 48). A random-effects model will be used to account for the expected heterogeneity in participant characteristics and methodologies between studies (47). For neuroimaging analyses, a cluster-level familywise-error-corrected threshold of *p* < 0.05 will be used to control for false positives, in accordance with best-practice recommendations (49). If sufficient data is not available for meta-analysis, a narrative synthesis of the results from included studies will be completed. Findings will be broadly organised by biological domain (i.e., molecular genetic, genomic, brain structural, and brain functional). For each biological domain, findings will be further organised according to the psychiatric phenotype (i.e., general and/or specific/lower-order latent dimensions) investigated in each analysis and according to the age group or developmental period being investigated. For longitudinal studies, evidence of biological factors that predict general psychopathology and specific/lower-order symptom dimensions will be separated from those that follow from general and specific/lower-order dimensions. A detailed summary of all significant findings will be provided.

### Quality assessment and risk of bias

Following data extraction, the quality of each included study will be assessed independently by NH using checklists from the Joanna Briggs Institute (50). Cross-sectional studies will be evaluated using the Checklist for Analytical Cross-Sectional Studies and longitudinal studies will be evaluated using the Checklist for Cohort Studies (50). The review will also include assessment of the overall quality of evidence at the outcome level, scored by NH using the Grades of Recommendation, Assessment, Development, and Evaluation (GRADE) system (51). The GRADE system provides a transparent framework for estimating the certainty with which review authors can assert that a given estimate of an effect is representative of the true effect (52). Level of certainty is assessed with reference to study design and within-study risk of bias, heterogeneity, indirectness of evidence, imprecision, and publication bias (51).

## Discussion

This paper outlines the protocol for a systematic review of studies investigating the biological correlates of latent transdiagnostic dimensions of psychopathology in general population samples across all age groups and developmental periods. Specifically, the review aims to integrate and assess evidence from studies investigating the molecular genetic, genomic, brain structural, and brain functional correlates of general psychopathology and specific/lower-order transdiagnostic symptom dimensions across the lifespan. This will be the first review to systematically examine the biological correlates of latent general psychopathology and specific/lower-order symptom dimensions and the first to characterise these relationships developmentally across the lifespan. The review is broadly intended to: integrate and assess the existing body of evidence; provide researchers with targets for investigating biological mechanisms and processes that are associated with latent transdiagnostic dimensional phenotypes at multiple levels of specificity; to identify targets for research investigating age- and developmentally specific relationships between biology and latent transdiagnostic dimensions of psychopathology; and to assess evidence regarding the temporal ordering of these relationships.

## Ethics statement

Ethical review and approval was not required for the study on human participants in accordance with the local legislation and institutional requirements. Written informed consent from the participants’ legal guardian/next of kin was not required to participate in this study in accordance with the national legislation and the institutional requirements.

## Author contributions

NH conceptualised the study, developed the methods and protocol, is the guarantor of the review, is responsible for data extraction, as well as quality and risk of bias assessments, and wrote the first draft of the manuscript. SL, MW, SR, and LM assisted with development of the methods and protocol. NH and SL were responsible for title and abstract screening and full-text screening. All authors revised and gave approval on the final manuscript.
