# Supplementary material for: Investigating the molecular genetic, genomic, brain structural, and brain functional correlates of latent transdiagnostic dimensions of psychopathology across the lifespan: Protocol for a systematic review and meta-analysis of cross-sectional and longitudinal studies in the general population
Source: Front Psychiatry. 2022 Nov 3;13:1036794. doi: 10.3389/fpsyt.2022.1036794 (PMC9669375; doi:10.3389/fpsyt.2022.1036794)
Supplement: Supplementary file 1 [file Data_Sheet_1.pdf]

SUPPLEMENTARY MATERIAL

Supplement Table 1

PRISMA-P (Preferred Reporting Items for Systematic review and Meta-Analysis Protocols) 2015 checklist: recommended items to address in a systematic review protocol[1]

Section and topic	Item No	Checklist item	Items Reported
**ADMINISTRATIVE INFORMATION**			
Title:			
Identification	1a	Identify the report as a protocol of a systematic review	☒
Update	1b	If the protocol is for an update of a previous systematic review, identify as such	☐
Registration	2	If registered, provide the name of the registry (such as PROSPERO) and registration number	☒
Authors:			
Contact	3a	Provide name, institutional affiliation, e-mail address of all protocol authors; provide physical mailing address of corresponding author	☒
Contributions	3b	Describe contributions of protocol authors and identify the guarantor of the review	☒
Amendments	4	If the protocol represents an amendment of a previously completed or published protocol, identify as such and list changes; otherwise, state plan for documenting important protocol amendments	☒
Support:			
Sources	5a	Indicate sources of financial or other support for the review	☒
Sponsor	5b	Provide name for the review funder and/or sponsor	☐
Role of sponsor or funder	5c	Describe roles of funder(s), sponsor(s), and/or institution(s), if any, in developing the protocol	☒
**INTRODUCTION**			
Rationale	6	Describe the rationale for the review in the context of what is already known	☒
Objectives	7	Provide an explicit statement of the question(s) the review will address with reference to participants, interventions, comparators, and outcomes (PICO)	☒
**METHODS**			
Eligibility criteria	8	Specify the study characteristics (such as PICO, study design, setting, time frame) and report characteristics (such as years considered, language, publication status) to be used as criteria for eligibility for the review	☒

Information sources	9	Describe all intended information sources (such as electronic databases, contact with study authors, trial registers or other grey literature sources) with planned dates of coverage	☒
Search strategy	10	Present draft of search strategy to be used for at least one electronic database, including planned limits, such that it could be repeated	☒
Study records:			
Data management	11a	Describe the mechanism(s) that will be used to manage records and data throughout the review	☒
Selection process	11b	State the process that will be used for selecting studies (such as two independent reviewers) through each phase of the review (that is, screening, eligibility and inclusion in meta-analysis)	☒
Data collection process	11c	Describe planned method of extracting data from reports (such as piloting forms, done independently, in duplicate), any processes for obtaining and confirming data from investigators	☒
Data items	12	List and define all variables for which data will be sought (such as PICO items, funding sources), any pre-planned data assumptions and simplifications	☒
Outcomes and prioritization	13	List and define all outcomes for which data will be sought, including prioritization of main and additional outcomes, with rationale	☒
Risk of bias in individual studies	14	Describe anticipated methods for assessing risk of bias of individual studies, including whether this will be done at the outcome or study level, or both; state how this information will be used in data synthesis	☒
Data synthesis	15a	Describe criteria under which study data will be quantitatively synthesised	☒
	15b	If data are appropriate for quantitative synthesis, describe planned summary measures, methods of handling data and methods of combining data from studies, including any planned exploration of consistency (such as I^2, Kendall's τ)	☒
	15c	Describe any proposed additional analyses (such as sensitivity or subgroup analyses, meta-regression)	☒
	15d	If quantitative synthesis is not appropriate, describe the type of summary planned	☒
Meta-bias(es)	16	Specify any planned assessment of meta-bias(es) (such as publication bias across studies, selective reporting within studies)	☐
Confidence in cumulative evidence	17	Describe how the strength of the body of evidence will be assessed (such as GRADE)	☒

Supplement Table 2

Embase search strategy

Database	Domain	Search terms
Embase	Latent dimensional models of psychopathology	**1. SH: exp factor analysis/ or principal component analysis/**<br>**OR**<br>**2.** general factor* or p-factor* or factor analys?s or "latent class analys?s" or "item response theory" or "factor mixture model*" or (transdiagnostic* adj4 (model* or structur* or dimension* or spectr*)) or (dimension* adj4 (model* or structur* or spectr*)) or (latent* adj4 (model* or structur* or dimension* or spectr*)) or (hierarch* adj4 (model* or structur* or dimension* or spectr*)) or CFA or PCA<br>**AND**<br>**3. SH: exp mental disease/ or exp psychiatry/**<br>**OR**<br>**4.** psychopatholog* or psychiatr* or internali?ing or externali?ing or thought disorder*
	Brain structural and brain functional neuroimaging studies	**5. SH: neuroimaging/ or nuclear magnetic resonance imaging/ or functional magnetic resonance imaging/ diffusion tensor imaging/ or functional connectivity/**<br>**OR**<br>**6.** (structur* connect* or function* connect* or (brain adj structur*) or (brain adj function*) or (neural adj (correlate* or structur* or substrate*)) or neuroimaging).mp
	Molecular genetic and genomic studies	**7. SH: Genetic analysis/ or behavior genetics/ or exp human genetics/ or genetic association/ or genetic variability/ or genetic correlation/ or genetic predisposition/ or pleiotropy/ or genetic risk score/ or genome analysis/ or genome-wide association study/ or single nucleotide polymorphism/**<br>**OR**<br>**8.** polygen* or pleiot* or "polygenic risk score*" or SNP* or GWA* or PGRS or PGS

Note 1. [Latent dimensional models of psychopathology] AND [brain structural OR brain functional] OR [molecular genetic OR Genomic]; SH, Subject Heading; CFA, Confirmatory Factor Analysis; PCA, Principal Component Analysis; SNP, Single Nucleotide Polymorphism; GWA, Genome-Wide Analysis; PGRS, Polygenic Risk Scores; PGS, Polygenic Scores.
Note 2. No limits were imposed on the date, language, or publication type for any of the three search strategies.
Note 3. The search strategy was executed across all three databases on 13 July 2021.
Note 4. This search returned 3581 articles (prior to de-duplication).

Supplement Table 3

PsycINFO search strategy

Database	Domain	Search terms
PsycINFO	Latent dimensional models of psychopathology	**1. SH: latent variables/ or exp factor analysis/ or latent class analysis/ or latent profile analysis/ or item response theory/ or principal component analysis/**
		**OR**
		**2.** (general factor* or p-factor* or factor analys?s or "latent class analys?s" or "item response theory" or "factor mixture model*" or (transdiagnostic* adj4 (model* or structur* or dimension* or spectr*)) or (dimension* adj4 (model* or structur* or spectr*)) or (latent* adj4 (model* or model* or structur* or dimension* or spectr*)) or (hierarch* adj4 (structur* or model* or dimension* or spectr*)) or CFA or PCA).mp
		**AND**
		**3. SH: exp psychopathology/ or exp psychiatry/ or exp mental disorders/**
		**OR**
		**4.** (psychopatholog* or psychiatr* or internali?ing or externali?ing or thought disorder*).mp
	Brain structural and brain functional neuroimaging studies	**5. SH: neuroimaging/ or exp magnetic resonance imaging/ or brain connectivity/**
		**OR**
		**6.** (structur* connect* or function* connect* or (brain adj structur*) or (brain adj function*) or (neural adj (correlate* or structur* or substrate*)) or neuroimaging).mp
	Molecular genetic and genomic studies	**7. SH: Genetics/ or Genes/ or Behavioral Genetics/ or Population Genetics/ or exp Genomics/ or Polymorphism/**
		**OR**
		**8.** (Polygen* or pleiot* or "single nucleotide polymorphism*" or SNP* or genetic association* or genome-wide association* or GWA* or "genetic risk score*" or "polygenic risk score*" or PGRS or PGS).mp

Note 1. [Latent dimensional models of psychopathology] AND [brain structural OR brain functional] OR [molecular genetic OR Genomic]; SH, Subject Heading; CFA, Confirmatory Factor Analysis; PCA, Principal Component Analysis; SNP, Single Nucleotide Polymorphism; GWA, Genome-Wide Analysis; PGRS, Polygenic Risk Scores; PGS, Polygenic Scores.
Note 2. No limits were imposed on the date, language, or publication type for any of the three search strategies.
Note 3. The search strategy was executed across all three databases on 13 July 2021.
Note 4. This search returned 985 articles (prior to de-duplication).

Supplement Table 4

MEDLINE search strategy

Database	Domain	Search terms
MEDLINE	Latent dimensional models of psychopathology	**1. SH: Latent class analysis/ or principal component analysis/ or factor analysis, statistical/**<br>**OR**<br>2. (general factor* or p-factor* or factor analys?s or latent class analys?s or item response theory or factor mixture model* or (transdiagnostic* adj4 (model* or structur* or dimension* or spectr*)) or (dimension* adj4 (model* or structur* or spectr*)) or (latent* adj4 (model* or structur* or dimension* or spectr*)) or (hierarch* adj4 (structur* or model* or dimension* or spectr*)) or CFA or PCA).mp<br>**AND**<br>**3. SH: Psychopathology/ or exp Psychiatry/ or exp Mental Disorders/**<br>**OR**<br>4. (Psychopathol* or psychiatr* or internali?ing or externali?ing or thought disorder*).mp
	Brain structural and brain functional neuroimaging studies	**5. SH: exp Neuroimaging/ or Magnetic Resonance Imaging/ or exp Diffusion Magnetic Resonance Imaging**<br>**OR**<br>6. (structur* connect* or function* connect* or (brain adj structur*) or (brain adj function*) or (neural adj (correlate* or structur* or substrate*)) or neuroimaging).mp
	Molecular genetic and genomic studies	**7. SH: Genetics/ or Human Genetics/ or Genetics, Behavioral/ or Genetics, Population/ or Genetic Predisposition to Disease/ or exp Genetic Association Studies/ or Polymorphism, Single Nucleotide/ or Genetic Variation/ or Genomics/ or Polymorphism, Genetic/ or Genetic Pleiotropy/**<br>**OR**<br>8. (Polygen* or pleiot* or SNP* or GWA* or genetic risk score* or polygenic risk score* or PGRS or PGS).mp

Note 1. [Latent dimensional models of psychopathology] AND [brain structural OR brain functional] OR [molecular genetic OR Genomic]; SH, Subject Heading; CFA, Confirmatory Factor Analysis; PCA, Principal Component Analysis; SNP, Single Nucleotide Polymorphism; GWA, Genome-Wide Analysis; PGRS, Polygenic Risk Scores; PGS, Polygenic Scores.
Note 2. No limits were imposed on the date, language, or publication type for any of the three search strategies.
Note 3. The search strategy was executed across all three databases on 13 July 2021.
Note 4. This search returned 1650 articles (prior to de-duplication).
